# PReS13-SPK-1587: New biologic targets for the future and basic science behind them

**DOI:** 10.1186/1546-0096-11-S2-I17

**Published:** 2013-12-05

**Authors:** B Prakken

**Affiliations:** 1 Pediatric Immunology, University Medical Centre Utrecht, Utrecht, Netherlands

## 

The treatment of paediatric rheumatic diseases has made great progress in the last decade. The introduction of the biologicals meant (after the introduction of MTX) the second therapeutic revolution. This together with many improvements in related areas has greatly improved the quality of life of patients with rheumatic diseases. At present however, despite the introduction of more biological agents directed at immune targets the progression has stalled. The next challenge will be to:

1) Better understand the immune pathogenesis underlying the heterogeneity of the disease.

2) Identify patients at risk at the onset of disease and stratify them according to risk factors.

3) Personalize treatment based on their risk profiling.

4) Develop strategies that are aimed not only at suppressing disease but also at maintaining disease remission after withdrawing immune suprresion.

Over the last years much progress has been made in all these area's. In this presentation we will discuss this progress, focusing on a better understanding of the mechanisms of immune tolerance, leading to new targets for intervention.

## Disclosure of interest

B. Prakken Grant/Research Support from: Dutch Arthritis Foundation.
